# Understanding Challenges and Emotions of Informal Caregivers of General Older Adults and People With Alzheimer Disease and Related Dementia: Comparative Study

**DOI:** 10.2196/54847

**Published:** 2025-02-28

**Authors:** Nova Mengxia Huang, Liang Ze Wong, Shirley S Ho, Bryan Timothy

**Affiliations:** 1 Nanyang Technological University Wee Kim Wee School of Communication and Information Singapore Singapore; 2 Institute of High Performance Computing Agency for Science, Technology and Research (A*STAR) Singapore Singapore; 3 National University of Singapore Yong Loo Lin School of Medicine Centre for Behavioural and Implementation Science Interventions Singapore Singapore

**Keywords:** informal caregivers, older adults, Alzheimer disease and related dementia, online support communities, Reddit

## Abstract

**Background:**

Faced with multiple challenges, informal caregivers often turn to online support communities for information and support. While scholarly attention has focused on experiences expressed by informal caregivers in these communities, how caregivers’ challenges and emotional expressions vary across different health contexts remains understudied.

**Objective:**

We aimed to examine and compare the challenges discussed by informal caregivers of general older adults and those of patients with Alzheimer disease and related dementia, as well as their emotional expressions, on Reddit. In addition, we examined how informal caregivers expressed their emotions in response to various challenges.

**Methods:**

We collected posts from 6 subreddits, including 3 subreddits on caregiving for older adults and 3 on caregiving for patients with Alzheimer disease and related dementia. Using topic modeling, we identified topics discussed by caregivers in the collected posts. We further used deep reading to contextualize these topics and understand the challenges behind them, conducted sentiment analysis to investigate their emotional expressions, and used Spearman rank-order correlation to examine the relationship between the obtained topics and emotions.

**Results:**

In total, 3028 posts were retrieved, including 1552 from older adult–related subreddits and 1476 from Alzheimer disease–related subreddits; 18 key topics were identified, with the most frequent topics being *expressing feelings* (2178/3028, 71.93%) and *seeking advice and support* (1982/3028, 65.46%). Other topics covered various challenges in caregiving, such as *duration of medical care* (1954/3028, 64.53%), *sleep and incontinence* (1536/3028, 50.73%)*, financial issues* (1348/3028, 44.52%), and *nursing home* (1221/3028, 40.32%). There was a positive, negligible correlation between *expressing feelings* and *seeking advice and support* (ρ=0.09, *P*<.001). Other topics also showed positive, negligible or weak correlations with these 2 topics but in distinct patterns. Posts from older adult–related subreddits were more focused on practical caregiving issues and *seeking advice and support*, whereas posts from Alzheimer disease–related subreddits emphasized health- and medical-related topics and *expressing feelings*. Caregivers in both contexts predominantly expressed negative emotions (older adults: 1263/1552, 81.38%; Alzheimer disease: 1247/1476, 84.49%), with caregivers in Alzheimer disease–related subreddits exhibiting slightly greater *fear* and *sadness* (*P*<.001). Specific challenges were significantly correlated with negative emotions: *duration of medical* *care* was positively, weakly correlated with *anger* (ρ=0.25, *P*<.001), *fear* (ρ=0.25, *P*<.001), and *sadness* (ρ=0.22, *P*<.001). *Medical appointments* were positively, negligibly correlated with *anger* (ρ=0.10, *P*<.001), *fear* (ρ=0.09, *P*<.001), and *sadness* (ρ=0.06, *P*<.001). *Sleep and incontinence* (ρ=0.14, *P*<.001) and *finances* (ρ=0.24, *P*<.001) were positively, weakly correlated with *anger*.

**Conclusions:**

By identifying the challenges and feelings expressed by caregivers for general older adults and caregivers for patients with Alzheimer disease and related dementia, our findings could inform health practitioners and policy makers in developing more targeted support interventions for informal caregivers in different contexts.

## Introduction

### Background

Many countries are faced with the challenge of an aging society. An aging society increases the likelihood that older adults live longer while coping with increased chronic diseases, thus underscoring the need for long-term care services [1,2]. Most older adults receive long-term care within their own residences, often relying on assistance from informal caregivers—family members, friends, and neighbors who provide unpaid care to people with health or functioning needs [1,3]. In the United States, 22.3% of adults provided such care or assistance in the past month, 10.4% of whom provided daily care for people with dementia or other cognitive impairment disorders [4]. Similar patterns are observed in some European countries, such as the United Kingdom and Germany [5].

Given the increasing prevalence of informal caregiving over the coming years, it is important to understand the challenges faced by informal caregivers of older adults [1,2]. Upon assuming the caregiving role, informal caregivers need to handle multiple tasks and responsibilities, including assisting with financial management, assisting with medical decision-making, and providing emotional support for their loved ones [1,2]. These responsibilities often conflict with caregivers’ existing roles and personal needs, leading to stressful experiences [1,2]. While caregiving may also bring positive experiences, such as finding satisfaction in caring for loved ones [6] and improving the caregivers’ sense of mastery [7,8], the demands often take a heavy toll on the caregivers’ health and well-being [9,10].

However, caregiving experiences may vary across different situations and contexts [11-13]. The Caregiver Stress Theory, a stress processing theory that is specifically created to account for variance in the stress experience of caregivers, specifies that factors related to the caregiving context, such as years of providing care, age, and the health status of care recipients, can influence every part of the stress process [10]. For instance, spouse caregivers experienced greater levels of stress caring for their loved ones as compared to adult child caregivers [14]. The theory also outlines 2 sources of caregivers’ stress, namely primary and secondary stressors [10]. Primary stressors refer to sources of stress that are directly related to acts of caregiving, such as the challenge from the care recipients’ diseases and symptoms and assisting with daily living tasks, and secondary stressors are those indirectly related to caregiving tasks, such as conflicts with other family members and social restrictions [10,15]. This framework thus provides a holistic understanding of the challenges that caregivers face in their caregiving journey and the factors affecting different caregiving experiences [10,15].

Particularly relevant to this study are the experiences of informal caregivers of people with Alzheimer disease and related dementia and general older adults. Compared to caregivers of general older adults, caregivers of people with Alzheimer disease and related dementia are likely to spend more time on caregiving responsibilities, ranging from bathing and feeding to assisting with toileting [16]. In addition, these caregivers perceive higher levels of physical and emotional burdens [17,18] and experience increased depression rates [16,19]. These differences highlight the importance of understanding caregiving experiences by considering the health status of care recipients [11,13]. Hence, this study aims to examine and compare the challenges faced by informal caregivers of people with Alzheimer disease and related dementia and those taking care of general older adults to advance the understanding of the factors affecting different caregiving experiences.

### Informal Caregivers in Online Support Communities

The rise of online support communities (OSCs) has provided informal caregivers of older adults with an avenue to connect and communicate with similar others [20,21]. These OSCs are embedded across computer-mediated platforms, such as Facebook, Reddit, and CaringBridge, allowing caregivers to share caregiving-related experiences and seek information and support [22-24]. Participation in OSCs has been shown to help caregivers solve caregiving-related issues, reduce feelings of isolation, improve self-efficacy in the process of caregiving [21,25], and improve overall well-being [26,27].

The interactions between caregivers in OSCs also offer valuable insights into their challenges [28,29] and their responses to said challenges [30]. Existing content analysis studies have provided evidence of the experiences of caregivers for either general older adults [30] or people with Alzheimer disease and related dementia [17,28,29,31]. They found that these caregivers often revealed caregiver burdens [17,24], exchanged social support [31], and engaged in stress-coping behaviors across OSCs [30]. However, limited research has directly compared the experiences of caregivers of people with Alzheimer disease and related dementia and general older adults revealed in OSCs [32]. Unpacking this is important as the knowledge can capture the complexity of the caregiving experience and the role of the caregiving context in shaping the unique challenges faced by caregivers. This suggests that a comparative study on the different experiences communicated by informal caregivers of general older adults and those caring for people with Alzheimer disease and related dementia in OSCs is necessary to better understand the unique needs for both groups.

### Emotional Expression in OSCs

Beyond sharing information about specific challenges, messages in OSCs can also reveal caregivers’ emotional experiences [33,34]. Caregiving can evoke a range of emotional responses among caregivers when facing challenges [35]. For example, anger may arise from the disruptive behaviors of the care recipient and strained relationships between the caregiver and the care recipient [35]. To cope with these emotions, caregivers often engage in the social sharing of emotions—openly communicating with others about emotion-eliciting events and their emotions (eg, joy, fear, anger, or sadness) [36]. Anonymous web-based communities such as Reddit provide a safe space for caregivers’ social sharing, where they may feel freer to share negative emotions compared to face-to-face settings or other identifiable groups, as these communities reduce social norms and alleviate privacy concerns [37,38]. Through this process, individuals can regulate their emotional experiences [39,40] and gain support and validation from others [41,42], which in turn reduces emotional distress [43].

While existing studies have examined informal caregivers’ emotional expressions in OSCs [18,30], 2 research gaps remain. First, little is known about how informal caregivers express emotions when responding to different caregiving challenges. Investigating the relationship between various caregiving challenges and emotional expressions can advance research on caregiver stress and coping. This knowledge, in turn, can inform more targeted support interventions to improve caregivers’ well-being. Second, it remains unclear how caregivers in different contexts, such as caregivers of general older adults and caregivers of people with Alzheimer disease and related dementia, express their emotions in OSCs. Faced with varying stressors and experiences, these 2 caregiver groups likely experience and express emotions differently. Comparing the patterns of emotional expression can contribute to a deeper understanding of the psychological needs of caregivers in different contexts, thus facilitating the development of targeted emotional and psychological interventions.

### Objectives

This study seeks to address these research gaps by analyzing discussions on Reddit among informal caregivers of older adults, including those taking care of general older adults and people with Alzheimer disease and related dementia. Reddit is a popular anonymous community that allows people to share experiences and seek support [44]. Many subreddits are established to support informal caregivers of older adults, including those especially for caregivers of people with Alzheimer disease and related dementia [17,24]. Therefore, Reddit provides a valuable source of data to investigate how different informal caregivers share their challenges and emotions in OSCs.

This study has 4 objectives. First, it aims to identify the topics discussed by informal caregivers of older adults on Reddit and the underlying challenges they face. Second, it aims to examine how these topics vary between caregivers of general older adults and caregivers of people with Alzheimer disease and related dementia. Third, it aims to investigate how informal caregivers of older adults express emotions in response to different topics and challenges. Fourth, it aims to compare the patterns of emotional expression between the 2 caregiver groups. Our research questions (RQs) are as follows:

- RQ1: What kinds of topics were discussed on Reddit by informal caregivers of older adults, and how do these topics reflect informal caregivers’ challenges?
- RQ2: What are the differences in topic distribution in subreddits for informal caregivers of general older adults and those caring for people with Alzheimer disease and related dementia?
- RQ3: What types of emotions were expressed by informal caregivers of older adults in relation to different topics on Reddit?
- RQ4: How do the emotions in Reddit posts differ across subreddits for informal caregivers of general older adults and those caring for people with Alzheimer disease and related dementia?

## Methods

### Overview

**Figure 1:**
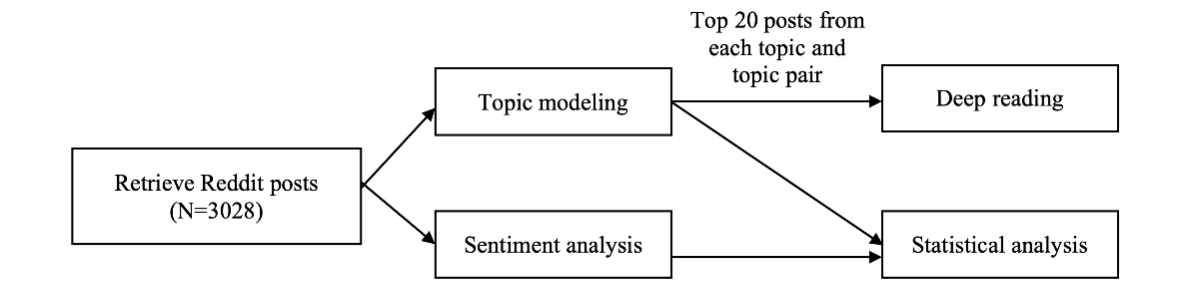
Research design.

To understand the experiences and feelings of informal caregivers of general older adults and people with Alzheimer disease and related dementia, we applied computer-assisted text analysis, including topic modeling and sentiment analysis, to posts from Reddit communities related to older adults and Alzheimer disease and related dementia caregiving. The computational methods were complemented by a qualitative deep reading, where we selected the top 20 posts from each topic and topic pair for qualitative analysis to provide deeper insight into the obtained topics [45]. In addition, we carried out correlational analysis within and between the topic scores and sentiment scores to identify associations between topics and sentiments. Figure 1 shows our research design.

### Data Collection

We focused on subreddits specifically designed to support informal caregivers of both general older adults and people with Alzheimer disease and related dementias. To identify relevant subreddits, we searched using the keywords “aging/older/elder” combined with “care/caregiver” for discussions about caring for older adults and “Alzheimer/dementia” combined with “care/caregiver” for those related to Alzheimer and dementia care. We then reviewed each subreddit’s description to ensure that it was a forum for informal caregivers of either the general older adult population or those with Alzheimer and related dementia. The selected subreddits for informal caregivers included r/AgingParents, r/eldercare, r/elderlycare, r/Alzheimers, r/dementia, and r/DementiaHelp. Other caregiver-related subreddits that were not specifically designed to support these caregiver populations were excluded from our data analysis for the purpose of this research.

We used the Reddit application programming interface (API) to retrieve top-level posts from these 6 subreddits. We retrieved up to 1000 of the most recent posts from each of these subreddits, on August 6, 2023, as the Reddit API only provides the 1000 most recent posts at the time of retrieval. For subreddits with fewer than 1000 posts at the time of retrieval, all posts were retrieved.

### Topic Modeling

We carried out topic modeling to uncover common topics that occur within this dataset. Topic modeling is a class of natural language processing methods that produces a set of topics from a text dataset, along with a table that indicates how strongly each of these topics occurs in each document.

We used a standard pipeline for topic modeling across the corpus of posts. First, we removed stop words, split the documents into terms with an n-gram range of 1 to 3 (thus allowing up to 3 words per term), and applied term frequency–inverse document frequency weighting to the document–term count matrix [46], which is a process that gives more weight to rarer but possibly more significant terms.

We then applied nonnegative matrix factorization (NMF), which factors the document-term matrix into a pair of document-topic and topic-term matrices [47]. The document-topic matrix shows which topics are present in each document, while the topic-term matrix shows how strongly each term is associated with each topic. We chose NMF rather than a clustering method like BERTopic [48] as we anticipated that each post could belong to multiple topics rather than to a single cluster. We also opted for NMF over latent Dirichlet allocation as NMF tends to give topics that are more meaningful and “more in line with human judgment” than latent Dirichlet allocation [49].

We carried out NMF with 10, 15, 20, and 25 topics. As the number of topics increased from 10 to 20, we observed additional meaningful topics appearing. However, increasing to 25 topics resulted in less coherent topics or splitting existing topics into subtopics. On this basis, we decided to take the results of NMF with 20 topics. From these 20 topics, we excluded 2 topics that consisted mainly of moderator posts about subreddit rules, resulting in a final 18 topics.

### Deep Reading

After topic modeling, we used a qualitative deep reading method, following the computation grounded theory by Nelson [50], to interpret the results. This deep reading involved closely examining representative texts for each topic identified from topic modeling and contextualizing key terms to uncover their meanings. By combining computational findings with this human-centered interpretation, we validated patterns identified from topic modeling and offered a comprehensive interpretation of the data [50].

The 2 authors, one specializing in social science and the other in natural language processing, independently reviewed the key terms and top 20 posts for each of the 18 topics to better understand what each topic was about and how the key terms were used within each topic. We individually named and defined each topic, identifying recurring themes. We then compared our findings and resolved any disagreement through discussion to clarify the meaning and context of each topic. For strongly correlated topic pairs, we examined representative posts that scored highly on both topics to better understand when and how these topics co-occur.

We selected the 20 posts that had the highest topic weight in each topic, as these posts contained the most key terms from an individual topic and were thus more representative of the topic [51]. Analyzing these top documents is a common practice in computational grounded theory, which enables a systematic and unbiased examination of the data [50]. This approach ensures that the selected documents accurately represent each topic while allowing for efficient analysis without the burden of interpreting the full dataset. Following prior studies analyzing the top 10 to 20 documents [50,52], we chose the top 20 posts of each topic for qualitative deep reading.

To ensure the rigor and trustworthiness of findings, this study used the interpretive paradigm to ensure the credibility, dependability, and confirmability of our analysis [53]. First, to enhance credibility (ie, the results of qualitative analysis adequately represent multiple realities), we discussed our data collection and analysis procedures with senior colleagues who had expertise in qualitative research. Second, dependability (ie, the consistency of data analysis) was achieved by documenting our thoughts during the deep reading process and engaging in frequent discussions to resolve any inconsistencies. Third, to ensure that researchers’ interpretations of data were nonprejudiced (ie, confirmability), we reflected critically on our own perspectives during the deep reading and included verbatim quotes to support the inferences presented in the results. These steps minimized researcher bias in handling data and enhanced the objectivity of our findings.

### Sentiment Analysis

We also used the CrystalFeel sentiment analysis API [54] to extract sentiment scores for each post. The CrystalFeel algorithm uses a mixture of lexicon- and word-embedding–based approaches to generate emotional intensity scores and has been validated on human-annotated test samples (achieving correlations of 0.765, 0.818, 0.788, and 0.765 with human annotations on fear, anger, joy, and sadness intensity, respectively) and used in multiple studies for sentiment analysis of social media posts [55,56]. We used CrystalFeel to generate continuous intensity scores (ranging from 0 to 1) for the 4 basic emotions (anger, fear, joy, and sadness), and an overall 5-point sentiment score ranging from −2 (very negative) to +2 (very positive).

### Statistical Analysis

To uncover associations between topics and emotions, we computed the Spearman rank correlation coefficient (ρ) among topic weights, and between topic weights and emotion scores. The Spearman rank correlation coefficient reflects the presence of a monotonic (but not necessarily linear) relationship between variables. We chose the Spearman rank correlation coefficient instead of the Pearson correlation coefficient because the distributions of topic weights resulting from NMF were nonnegative and highly skewed toward 0 [57]. The correlations between topic weights in this study reflect the degree of co-occurrence of topics across posts: a higher correlation between two topics indicates that those two topics are more likely to occur within the same post.

Following commonly used guidelines for interpretation (eg, [58]), correlations with a magnitude between 0 and 0.09 were considered negligible, while correlations with a magnitude between 0.1 and 0.39 were considered weak. Note that in our sample, correlations of magnitude >0.06 were significant at *P*<.001.

### Ethical Considerations

Before collecting data for this content analysis study, we obtained approval from the Nanyang Technological University’s Integrity Review Board (IRB-2023-258) under the exempt review category. Secondary research on existing or publicly accessible datasets, such as our study on Reddit posts, qualifies for this category. Additional informed consent from human subjects is not required for secondary analyses of research data. In compliance with the Integrity Review Board guidelines, we also protected the privacy and confidentiality of individuals by anonymizing users whose data were being analyzed and removing any identifiable information from the posts. No compensation was offered, as this study did not involve direct participation or recruitment of individuals. By strictly following these guidelines, we ensured that this study does not lead to adverse outcomes for human subjects.

## Results

### Dataset Description

In total, 3028 posts were retrieved from Reddit. Table 1 shows the breakdown of the number of posts retrieved from each subreddit.

**Table 1:** Number of posts retrieved from each subreddit (N=3028).

Subreddits		Posts, n (%)
**Older adults–related** **posts**		1552 (51.25)
	r/AgingParents	937 (30.94)
	r/eldercare	490 (16.18)
	r/elderlycare	125 (4.13)
**Dementia-related posts**		1476 (48.75)
	r/Alzheimers	916 (30.25)
	r/dementia	547 (18.06)
	r/DementiaHelp	13 (0.43)

### Topic Descriptions and Interpretation (RQ1)

Through topic modeling and deep reading, we identified 18 topics discussed by informal caregivers and the underlying challenges they faced (Table 2). By reviewing the key terms and top 20 posts within each topic, we named and described each topic and grouped the topics into 7 overarching themes. Representative posts within each topic are provided in Multimedia Appendix 1.

**Table 2:** Topic labels, key terms, and descriptions from each topic, along with the topic counts and frequency^a^ (N=3028).

Topic, topic name, and description				Topic count, n (%)		Key terms
**Theme 1: expressing feelings**						
	T1	Expressing feelings: discussing feelings about circumstances, including sadness, guilt, helplessness, frustration, stress, and anger.	2178 (71.93)		“just, like, feel, know, things, really, time, feel like, want, does, life, think, make, say, day, hard, people feeling”	
**Theme 2: seeking advice and support**						
	T4	Seeking advice and support: asking for advice in dealing with circumstances, often in the form of questions.	1982 (65.46)		“help, advice, need, does, house, needs, getting, need help, refuses, family, lives, want, health, trying, live, suggestions, looking, support”	
**Theme 3: health and medical concerns**						
	T2	Duration of medical care: discussing the care recipient’s health or behavioral issues, or the need for long-term medical care.	1954 (64.53)		“did, said, hospital, told, days, got, doctor, ago, went, day, today, weeks, week, going, months, called, hospice, just, years, took”	
	T9	Dementia: seeking or providing information about dementia	1605 (53.01)		“dementia, family, people, loved, diagnosed, people dementia, vascular dementia, vascular, caregivers, person, diagnosed dementia”	
	T7	Alzheimer: seeking or providing information about Alzheimer disease	1584 (52.31)		“alzheimer, early, onset, early onset, disease, diagnosed, onset alzheimer, early onset alzheimer, diagnosis, years, stage, experience, memory”	
	T3	Aging and declining health of parents: discussing the aging and the declining health status of parents	1572 (51.92)		“parents, aging, parent, health, life, years, live, aging parents, care, elderly, old, support, time, don, kids, issues, love, age, mental, situation”	
	T8	Sleep and incontinence: discussing sleep problems in general, or specifically incontinence.	1536 (50.73)		“bed, sleep, night, day, time, hours, door, room, toilet, does, bathroom, sleeping, gets, goes, sleeps, walk, wake, fall, diaper, incontinence”	
	T12	Medical appointments: discussing different types of medical appointments and experiences with such appointments	1082 (35.73)		“appointment, make, doctor, make appointment, bad, make doctor, said make appointment, memory gotten really, just yelled, make”	
**Theme 4: care facilities and services**						
	T6	Memory care facility: discussing memory care facilities for the care recipient, often seeking recommendations for such facilities, or describing their experiences with facilities	1396 (46.10)		“care, memory care, memory, facility, care facility, memory care facility, facilities, needs, need, area, elder, home care, residents, unit”	
	T11	Nursing home: discussing nursing home for the care recipient, often seeking recommendations for such facilities, or describing their experiences with facilities	1221 (40.32)		“home, nursing, nursing home, care, hospital, home care, medicaid, homes, rehab, nursing homes, home health, work, health, pay”	
	T10	Assisted-living facility: discussing assisted-living facilities, often seeking recommendations for such facilities, or describing their experiences with facilities.	1160 (38.31)		“living, assisted living, assisted, facility, living facility, assisted living facility, independent living”	
**Theme 5: care recipients and family relationships**						
	T14	Parents (mom): mentioning issues in dealing with mom	1571 (51.88)		“mom, sister, caregiver, dog, live, brother, care mom, care, mom, job, dad, mom does, best, mom diagnosed, taking, getting, don, moms, work, hours”	
	T16	Parents (mother): mentioning issues in dealing with mother	1534 (50.66)		“mother, sister, father, years, time, year, law, away, lives, old, year old, died, siblings, cat, family, passed, brother, care mother, come, mother law”	
	T15	Parents (dad): mentioning issues in dealing with dad	1525 (50.36)		“dad, mum, brother, home, years, just, want, does, months, help dad, ago, mom dad, dad does, live, care dad, angry, old, father, point, away”	
	T18	Grandparents: mentioning grandparents in general, or specific grandparents, often talking about their health problems.	1178 (38.90)		“grandma, grandpa, grandparents, aunt, grandmother, family, uncle, want, know, care grandma, does, live, home, help grandma”	
**Theme 6: financial, legal, and insurance issues**						
	T5	Finances: discussing finance-related matters, such as bills or handling of the ward’s assets. Many also mention power of attorney.	1348 (44.52)		“money, account, bank, poa, card, pay, house, attorney, credit, financial, power, finances, power attorney, trust”	
	T19	Long-term care insurance: discussing long-term care insurance, often asking for recommendations for long-term care insurance providers.	1182 (39.04)		“term, long term, long-term care, term care, long, insurance, care, term care insurance, care insurance, insurance, policy”	
**Theme 7: technological issues**						
	T13	Cell phone and devices: discussing phones and related devices, descriptions of difficulties encountered by the care recipient in using such devices, and requests for recommendations of devices.	1605 (53.01)		“phone, use, hearing, alert, elderly, looking, recommendations, device, app, cell, aids, hearing aids, watch, cell phone, devices”	

^a^Frequency refers to the percentage of posts with nonzero weights for that topic.

Theme 1 (ie, topic 1), named *expressing feelings*, accounted for the largest proportion of the topic distribution. Posts on this topic mentioned the posters’ feelings about circumstances, usually including negative feelings, such as sadness, guilt, helplessness, and frustration. The second most influential theme, theme 2 (ie, topic 4), was *seeking advice*. Posts under this topic focused on asking for advice in dealing with problems, often in the form of questions.

Other themes covered a wide variety of caregiving-related issues. Theme 3 (ie, topics 2, 3, 7, 8, 9, and 12) discussed health and medical issues related to the care recipient, including duration of medical care, dementia, Alzheimer, aging and declining health of parents, sleep and incontinence, and medical appointments. Theme 4 (ie, topics 6, 10, and 11) was about different types of care facilities and services, including memory care facilities, nursing homes, and assisted-living facilities. Theme 5 (ie, topics 14, 15, 16, and 18) focused on care recipients and family relationships, talking about either the posters’ mother, father, or grandparents. Theme 6 (ie, topics 5 and 6) was about financial, legal, and insurance issues, including finance-related issues such as bills or assets, often mentioning power of attorney, and long-term care insurance. Theme 7 (ie, topic 13) discussed technological issues, especially cell phones and hearing aid devices.

We also explored the relationships between different topics to have a more detailed understanding of these topics (Table 3). All correlations between topics were negligible (0-0.09 in magnitude) or weak (0.1-0.39 in magnitude).

**Table 3:** Spearman rank correlations between topics.

Theme and topic				1		2		3												4						5								6				7
			T1		T4		T2		T9		T7		T3		T8		T12		T6		T11		T10		T14		T16		T15		T18		T5		T19		T13	
**1**																																						
	**T1** **.** **Expressing feelings**																																					
		ρ	1		*0.09^a^*		*0.18*		0.05		*0.07*		*0.10*		*0.11*		*0.09*		*0.07*		0.04		0.00		*0.15*		*0.14*		*0.19*		*0.14*		0.02		0.01		−0.03	
		*P* value	—^b^		<.001		<.001		0.01		<.001		<.001		<.001		<.001		<.001		0.03		0.80		<.001		<.001		<.001		<.001		0.33		0.70		0.15	
**2**																																						
	**T4** **.** **Seeking advice and support**																																					
		ρ	*0.09*		1		0.01		0.00		−*0.07*		*0.13*		*0.12*		*0.11*		*0.08*		*0.12*		*0.09*		0.05		*0.12*		*0.08*		*0.11*		*0.13*		*0.09*		0.01	
		*P* value	<.001		—		0.63		0.94		<.001		<.001		<.001		<.001		<.001		<.001		<.001		0.01		<.001		<.001		<.001		<.001		<.001		0.74	
**3**																																						
	**T2** **.** **Duration of medical care**																																					
		ρ	*0.18*		0.01		1		0.04		*0.06*		0.00		*0.18*		*0.08*		*0.09*		*0.09*		0.04		*0.13*		*0.16*		*0.20*		*0.07*		0.02		0.04		−*0.08*	
		*P* value	<.001		0.63		—		0.02		<.001		0.99		<.001		<.001		<.001		<.001		0.02		<.001		<.001		<.001		<.001		0.26		0.04		<.001	
	**T9** **.** **Dementia**																																					
		ρ	0.05		0.00		0.04		1		*0.19*		0.01		−0.03		*0.08*		*0.07*		−0.05		−0.04		−0.04		*0.07*		−0.01		*0.06*		−0.06		0.01		0.00	
		*P* value	0.01		0.94		0.02		—		<.001		0.61		0.11		<.001		<.001		0.01		0.03		0.04		<.001		0.48		<.001		0.001		0.78		0.95	
	**T7** **.** **Alzheimer**																																					
		ρ	*0.07*		−*0.07*		*0.06*		*0.19*		1		−0.02		−0.05		*0.06*		−0.03		−*0.15*		−*0.10*		−0.02		*0.10*		0.03		0.04		−*0.15*		0.00		0.00	
		*P* value	<.001		<.001		<.001		<.001		—		0.30		0.01		<.001		0.08		<.001		<.001		0.31		<.001		0.15		0.05		<.001		0.81		0.90	
	**T3** **.** **Aging and declining health of parents**																																					
		ρ	*0.10*		*0.13*		0.00		0.01		−0.02		1		−*0.10*		0.02		*0.08*		*0.08*		*0.11*		*0.15*		*0.17*		*0.13*		−0.02		*0.14*		*0.14*		0.02	
		*P* value	<.001		<.001		0.99		0.61		0.30		—		<.001		0.17		<.001		<.001		<.001		<.001		<.001		<.001		0.36		<.001		<.001		0.36	
	**T8** **.** **Sleep and incontinence**																																					
		ρ	*0.11*		*0.12*		*0.18*		−0.03		−0.05		−*0.10*		1		0.01		0.00		*0.08*		0.02		0.02		0.01		*0.10*		*0.07*		−0.05		−0.01		0.05	
		*P* value	<.001		<.001		<.001		0.11		0.01		<.001		—		0.59		0.97		<.001		0.27		0.21		0.70		<.001		<.001		0.004		0.69		0.003	
	**T12** **.** **Medical appointments**																																					
		ρ	*0.09*		*0.11*		*0.08*		*0.08*		*0.06*		0.02		0.01		1		0.04		−0.05		−0.02		0.03		0.03		*0.07*		0.02		0.06		−0.02		−0.01	
		*P* value	<.001		<.001		<.001		<.001		<.001		0.17		0.59		—		0.02		0.01		0.26		0.17		0.14		<.001		0.33		0.001		0.33		0.73	
**4**																																						
	**T6** **.** **Memory care facility**																																					
		ρ	*0.07*		*0.08*		*0.09*		*0.07*		−0.03		*0.08*		0.00		0.04		1		*0.22*		*0.14*		*0.08*		*0.08*		0.02		0.00		*0.06*		*0.28*		−0.04	
		*P* value	<.001		<.001		<.001		<.001		0.08		<.001		0.97		0.02		—		<.001		<.001		<.001		<.001		0.29		0.85		<.001		<.001		0.02	
	**T11** **.** **Nursing home**																																					
		ρ	0.04		*0.12*		*0.09*		−0.05		−*0.15*		*0.08*		*0.08*		−0.05		*0.22*		1		*0.18*		*0.13*		0.06		*0.07*		*0.07*		*0.10*		*0.18*		−*0.06*	
		*P* value	0.03		<.001		<.001		0.01		<.001		<.001		<.001		0.01		<.001		—		<.001		<.001		0.001		<.001		<.001		<.001		<.001		<.001	
	**T10** **.** **Assisted-living facility**																																					
		ρ	0.00		*0.09*		0.04		−0.04		−*0.10*		*0.11*		0.02		−0.02		*0.14*		*0.18*		1		*0.08*		*0.07*		0.01		0.01		*0.19*		*0.11*		0.00	
		*P* value	0.80		<.001		0.02		0.03		<.001		<.001		0.27		0.26		<.001		<.001		—		<.001		<.001		0.74		0.47		<.001		<.001		0.97	
**5**																																						
	**T14** **.** **Parents (mom)**																																					
		ρ	*0.15*		0.05		*0.13*		−0.04		−0.02		*0.15*		0.02		0.03		*0.08*		*0.13*		*0.08*		1		0.03		0.15		0.00		*0.07*		*0.07*		−*0.12*	
		*P* value	<.001		0.01		<.001		0.04		0.31		<.001		0.21		0.17		<.001		<.001		<.001		—		0.06		<.001		0.83		<.001		<.001		<.001	
	**T16** **.** **Parents (mother)**																																					
		ρ	*0.14*		*0.12*		*0.16*		*0.07*		*0.10*		*0.17*		0.01		0.03		*0.08*		0.06		*0.07*		0.03		1		0.06		*0.09*		*0.12*		0.06		−0.05	
		*P* value	<.001		<.001		<.001		<.001		<.001		<.001		0.70		0.14		<.001		0.001		<.001		0.06		—		0.002		<.001		<.001		0.002		0.004	
	**T15** **.** **Parents (dad)**																																					
		ρ	*0.19*		*0.08*		*0.20*		−0.01		0.03		*0.13*		*0.10*		*0.07*		0.02		*0.07*		0.01		*0.15*		0.06		1		0.00		*0.06*		0.01		−*0.07*	
		*P* value	<.001		<.001		<.001		0.48		0.15		<.001		<.001		<.001		0.29		<.001		0.74		<.001		0.002		—		0.79		<.001		0.57		<.001	
	**T18** **.** **Grandparents**																																					
		ρ	*0.14*		*0.11*		*0.07*		*0.06*		0.04		−0.02		*0.07*		0.02		0.00		*0.07*		0.01		0.00		*0.09*		0.00		1		0.03		−*0.06*		−0.03	
		*P* value	<.001		<.001		<.001		<.001		0.05		0.36		<.001		0.33		0.85		<.001		0.47		0.83		<.001		0.79		—		0.15		<.001		0.06	
**6**																																						
	**T5** **.** **Finances**																																					
		ρ	0.02		*0.13*		0.02		−0.06		−*0.15*		*0.14*		−0.05		0.06		*0.06*		*0.10*		*0.19*		*0.07*		*0.12*		*0.06*		0.03		1		*0.10*		0.02	
		*P* value	0.33		<.001		0.26		0.001		<.001		<.001		0.004		0.001		<.001		<.001		<.001		<.001		<.001		<.001		0.15		—		<.001		0.36	
	**T19** **.** **Long-term care insurance**																																					
		ρ	0.01		*0.09*		0.04		0.01		0.00		*0.14*		−0.01		−0.02		*0.28*		*0.18*		*0.11*		*0.07*		0.06		0.01		−*0.06*		*0.10*		1		0.01	
		*P* value	0.70		<.001		0.04		0.78		0.81		<.001		0.69		0.33		<.001		<.001		<.001		<.001		0.002		0.57		<.001		<.001		—		0.66	
**7**																																						
	**T13** **.** **Cell phone and devices**																																					
		ρ	−0.03		0.01		−*0.08*		0.00		0.00		0.02		0.05		−0.01		−0.04		−*0.06*		0.00		−*0.12*		−0.05		−*0.07*		−0.03		0.02		0.01		1	
		*P* value	0.15		0.74		<.001		0.95		0.90		0.36		0.003		0.73		0.02		<.001		0.97		<.001		0.004		<.001		0.06		0.36		0.66		—	

^a^Correlations with *P*<.001 are italicized according to the strength and magnitude of the correlations.

^b^Not applicable.

There was a positive negligible correlation between the two most frequently discussed topics, *seeking advice and support* and *expressing feelings* (ρ=0.09, *P*<.001). Other topics also showed positive, negligible or weak correlations with these two topics but in distinct patterns. Topics related to health and medical concerns, such as *duration of medical care* (ρ=0.18, *P*<.001), *aging and declining health of parents* (ρ=0.10, *P*<.001), *sleep and incontinence* (ρ=0.11, *P*<.001), and *medical appointments* (ρ=0.09, *P*<.001), were positively, weakly or negligibly correlated with *expressing feelings*. In addition, 3 of these topics—*aging and declining health of parents*, *sleep and incontinence*, and *medical appointments*—were also positively, weakly correlated with *seeking advice and support*, with Spearman ρ values of 0.13, 0.12, and 0.11, respectively (all *P*<.001).

Topics related to care facilities and services, including *nursing home* (ρ=0.12, *P*<.001) and *assisted-living facility* (ρ=0.09, *P*<.001), as well as financial topics such as *finances* (ρ=0.13, *P*<.001) and *long-term care insurance* (ρ=0.09, *P*<.001), were positively, weakly or negligibly correlated with *seeking advice and support*.

To interpret the challenges caregivers faced, we analyzed representative posts that scored high in the above topic combinations by deeply reading. When discussing the *duration of medical care* of the care recipient, such as the care recipient’s health or behavioral issues, posters usually expressed negative feelings, such as helplessness and frustration, as a means of venting or seeking comfort. For example, one caregiver shared his fear and uncertainty following his grandmother’s abnormal behavioral problems due to dementia:

So my grandma (90) was quite healthy in body and mind until this April... she started showing signs of dementia: More severe forgetfulness, language problems, episodes where she didn’t seem like she is “now”...I have no experience or expertise with dementia and frankly I am very scared right now...It just seemed so sudden and such a radical change.

In contrast, posts discussing more practical issues in caregiving, including health and social services, insurance, and financial topics, tended to seek advice or help. For example, a caregiver expressed the need to secure an affordable nursing home:

The hospital called my grandmother and said her husband was being discharged and to pick him up. He has dementia and diabetes... how can we find a home he can afford when his income is below the “needed rate”? We’ve called many various services and no one seems to be able to help or give guidance.

Caregivers discussing health and medical issues, including aging and declining health of parents, sleep and incontinence, and medical appointments, tended to express both feelings and a need for advice. For example, posts about sleep and incontinence usually directly sought advice to solve the problem while also revealing the emotional toll on caregivers:

I help (pretty much am the main) care for my MIL...The problem is bedtime. She doesn’t like to be alone. She wanders around every 15 minutes and will not go to bed...It’s cold and I’m tired and miserable and I feel so guilty for hiding like this...Any suggestions what I can do to get space so I can sleep? I’m starting to lose my sanity.

### Topic Distribution Comparison (RQ2)

**Figure 2:**
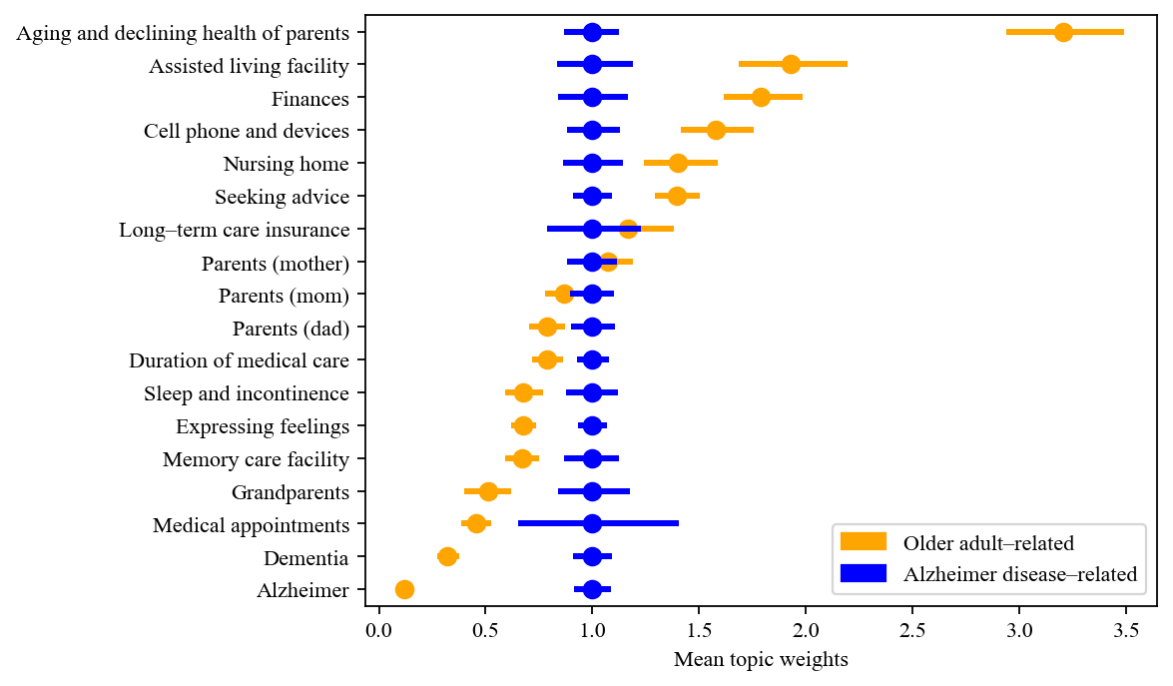
Comparison of mean topic weights between older adult– and Alzheimer disease–related subreddits. Horizontal bars represent 95% CIs for the mean topic weight. Weights for each topic are normalized such that the mean topic weight for Alzheimer disease–related subreddits is 1 and sorted according to the normalized mean topic weight for older adult–related subreddits.

Comparing the topics discussed in subreddits for informal caregivers of general older adults and those caring for people with Alzheimer disease and related dementia, we found that while all topics were present in posts from both older adult–related and Alzheimer disease–related subreddits, certain topics were more prevalent in one community than the other (Figure 2)*.* Posts from older adult–related subreddits tended to be *seeking advice and support.* Compared with Alzheimer disease–related subreddits, they showed greater interest in talking about *aging and declining health of parents*, and care facility–related topics, such as *assisted-living facilities and nursing homes.* In addition*,* they also focused more on financial, legal, insurance, and technical issues (ie, *finances*, *long-term care insurance*, and *cell phones and devices)*.

However, posts from Alzheimer disease–related subreddits tended to be *expressing feelings*. They showed greater interest in other health- and medical-related topics, such as *duration of medical care*, *sleep and incontinence*, and *medical appointments*. They also tended to talk more about *dementia*, *Alzheimer*, and *memory care facilities—*the topics in relation to Alzheimer and related dementia.

### Emotions in Relation to Topics (RQ3)

We analyzed the relationships between 18 topics and emotions obtained from sentiment analysis to understand how caregivers expressed emotions in response to various topics. The results (Table 4) indicated that most topics were positively but weakly correlated with negative emotions and sentiments. The only topic that had a positive, weak correlation with positive sentiment was *cell phones and devices* (ρ=0.20, *P*<.001).

The topic *duration of medical care* was positively, weakly correlated with a set of negative emotions, including *anger* (ρ=0.25, *P*<.001), *fear* (ρ=0.25, *P*<.001), and *sadness* (ρ=0.11, *P*<.001). Similarly, *medical appointments* were positively, negligibly correlated with these emotions, including *anger* (ρ=0.10, *P*<.001), *fear* (ρ=0.09, *P*<.001), and *sadness* (ρ=0.06, *P*<.001). The topics *sleep and incontinence* (ρ=0.14, *P*<.001) and *finances* (ρ=0.24, *P*<.001) were both positively, weakly correlated with *anger*.

While the topic of *Alzheimer* was negatively, weakly correlated with *anger* (ρ=−0.10, *P*<.001), it was positively, weakly correlated with *fear* (ρ=0.10, *P*<.001). The topic of *dementia* was positively, weakly correlated with *fear* (ρ=0.12, *P*<.001) and *sadness* (ρ=0.09, *P*<.001).

**Table 4:** Spearman rank correlationa between topic weights and emotion intensity scores.

Theme and topic				Sentiment		Joy		Anger		Fear		Sadness			
**1**															
	Express feelings		ρ		−*0.20*		−*0.12*		*0.17*		*0.23*		*0.29*		
			*P* value		<.001		<.001		<.001		<.001		<.001		
**2**															
	Seeking advice and support		ρ		−*0.11*		−*0.10*		*0.10*		*0.08*		*0.07*		
			*P* value		<.001		<.001		<.001		<.001		<.001		
**3**															
	Duration of medical care		ρ		−*0.18*		−*0.11*		*0.25*		*0.25*		*0.22*		
			*P* value		<.001		<.001		<.001		<.001		<.001		
	Dementia		ρ		−0.04		−*0.08*		0.00		*0.12*		*0.09*		
			*P* value		0.03		<.001		0.83		<.001		<.001		
	Alzheimer		ρ		0.01		0.00		−*0.10*		*0.10*		0.06		
			*P* value		0.74		0.83		<.001		<.001		0.002		
	Aging and declining health of parents		ρ		−0.03		0.01		0.02		0.06		*0.10*		
			*P* value		0.08		0.51		0.37		0.002		<.001		
	Sleep and incontinence		ρ		−*0.12*		−0.03		*0.14*		0.06		0.01		
			*P* value		<.001		0.11		<.001		0.001		0.66		
	Medical appointments		ρ		−*0.08*		−*0.11*		*0.10*		*0.09*		*0.06*		
			*P* value		<.001		<.001		<.001		<.001		<.001		
**4**															
	Memory care facility		ρ		−0.01		0.00		0.01		0.06		*0.07*		
			*P* value		0.78		0.91		0.75		0.002		<.001		
	Nursing home		ρ		−0.03		0.00		0.05		*0.09*		*0.09*		
			*P* value		0.08		0.94		0.01		<.001		<.001		
	Assisted-living facility		ρ		−0.04		−0.03		0.05		0.04		0.03		
			*P* value		0.03		0.11		0.003		0.04		0.14		
**5**															
	Parents (mom)		ρ		−*0.10*		−0.04		*0.12*		*0.13*		*0.15*		
			*P* value		<.001		0.05		<.001		<.001		<.001		
	Parents (mother)		ρ		−*0.13*		−*0.10*		*0.13*		*0.16*		*0.18*		
			*P* value		<.001		<.001		<.001		<.001		<.001		
	Parents (dad)		ρ		−*0.17*		−*0.15*		*0.17*		*0.15*		*0.16*		
			*P* value		<.001		<.001		<.001		<.001		<.001		
	Grandparents		ρ		−0.04		0.02		0.00		0.02		0.06		
			*P* value		0.01		0.26		0.87		0.22		0.002		
**6**															
	Finances		ρ		−*0.09*		−*0.13*		*0.24*		−0.03		0.00		
			*P* value		<.001		<.001		<.001		0.08		0.80		
	Long-term care insurance		ρ		0.02		0.00		−0.02		0.02		0.01		
			*P* value		0.30		0.83		0.38		0.18		0.57		
**7**															
	Cell phone and devices		ρ		*0.20*		*0.16*		−*0.18*		−*0.33*		−*0.33*		
			*P* value		<.001		<.001		<.001		<.001		<.001		

^a^Correlations with *P*<.001 significance are italicized.

### Emotions Comparisons (RQ4)

Approximately >70% (older adults: 1117/1552, 71.97%; Alzheimer disease: 1095/1476, 74.19%) of posts from both the Alzheimer disease– and older adult–related subreddits expressed an overall negative sentiment, with an additional 10% (older adults: 146/1552, 9.41%; Alzheimer disease: 152/1476, 10.30%) expressing a very negative sentiment (Table 5). In contrast, approximately 10% (older adults: 184/1552, 11.86%; Alzheimer disease: 138/1476, 9.35%) of posts expressed positive sentiments, and very few expressed very positive sentiments. The distribution of overall sentiment scores was similar between the 2 subreddit types.

**Table 5:** Distribution of sentiments across Alzheimer disease–related and older adult–related subreddits.

	Number of posts, n (%)	
	Alzheimer disease–related subreddits (n=1476)	Older adult–related subreddits (n=1552)
Very positive	2 (0.14)	2 (0.13)
Positive	138 (9.35)	184 (11.86)
Neutral	89 (6.03)	103 (6.64)
Negative	1095 (74.19)	1117 (71.97)
Very negative	152 (10.30)	146 (9.41)

Regarding the expression of specific emotions, *joy* was much less present in both types of subreddits compared with negative emotions (Table 6). In addition, there was slightly more *fear* and *sadness* expressed in the Alzheimer disease–related subreddits than in the older adult–related ones.

**Table 6:** Comparison of emotion values across Alzheimer disease– and older adult–related subreddits^a^.

	Emotion scores, mean (SD)	
	Alzheimer disease–related subreddits	Older adult–related subreddits
Fear^b^	0.51 (0.09)	0.48 (0.10)
Sadness^b^	0.49 (0.08)	0.47 (0.08)
Anger	0.46 (0.07)	0.46 (0.08)
Joy	0.26 (0.08)	0.26 (0.08)

^a^The value of each emotion score is a continuous variable ranging from 0 (ie, a message not expressing the emotion at all) to 1 (ie, a message expresses an extremely high intensity of the emotion).

^b^The differences were significant at *P*<.001.

## Discussion

### Principal Findings

To provide insights into the challenges and emotional experiences of informal caregivers of older adults, this study combined computer-assisted text analysis (ie, topic modeling and sentiment analysis) with qualitative analysis (ie, deep reading) to analyze the topics, challenges, and emotions expressed by informal caregivers of general older adults and people with Alzheimer disease and related dementia on Reddit. We identified 18 topics from informal caregivers’ posts, with posts predominantly focusing on expressing personal feelings and seeking advice and support. These posts revealed various challenges faced by informal caregivers, including managing care recipients’ health and medical issues and other practical issues. When discussing these caregiving-related challenges, informal caregivers either expressed their negative feelings or sought help from other forum members.

Our analysis also revealed differences in topics and emotions expressed by users in subreddits for informal caregivers of general older adults and those for people with Alzheimer disease and related dementia. While caregivers of general older adults focused more on practical topics, such as care facilities, caregivers of people with Alzheimer disease and related dementia discussed more about their loved one’s health and medical problems, such as dementia disease. In addition, the posts of caregivers of people with Alzheimer disease and related dementia expressed higher levels of negative emotions, such as fear and anger, although posts from both subreddits revealed an overall negative sentiment. Notably, we found that informal caregivers had distinct emotional expressions when discussing different topics. Posts about the duration of medical care were positively weakly correlated with a complex set of negative emotions, including anger, fear, and sadness. When talking about sleep, incontinence, and financial issues, informal caregivers tended to express anger, while Alzheimer disease–related topics were positively but weakly correlated with fear. The findings indicate a nuanced relationship between caregivers’ challenges and their emotional expressions.

### Comparison With Previous Works

Existing studies have provided evidence that caregivers often turned to web-based communities, such as Facebook [29] and blogs [28], to share caregiving-related experiences and seek desired information. Similarly, our study found that expressing feelings and seeking advice were predominant topics on subreddits for informal caregivers. According to social support research, both expressing feelings and seeking advice are effective strategies for eliciting social support from others. Expressing feelings, particularly negative ones, may signal a need for emotional support [59,60]. Therefore, the findings suggest that Reddit serves as a platform where caregivers seek social support to cope with caregiving challenges [61].

By combining topic modeling and deep reading, this study also identified specific challenges revealed in web-based discussions among informal caregivers, including managing the health and medical issues of their loved ones, dealing with family relationships, and handling practical caregiving tasks, such as finding appropriate care facilities. These findings align with previous research on informal caregivers’ web-based self-disclosure [17,29,62]. For example, analysis of Facebook posts by Bachmann [29] found that caregivers of patients with Alzheimer disease face diverse challenges concerning extended activities of daily living, such as dealing with sleeping disorders, selecting health facilities, and managing medical appointments and tests. The results of this study also complement self-reported data from informal caregivers of older adults [63,64]. For instance, a qualitative study in 4 European countries found that informal caregivers reported facing multiple challenges and needs related to the management of care for older adults [63]. Consistent with these studies, our findings demonstrate the multiple challenges faced by informal caregivers and highlight the need for further research to address their specific needs in caregiving.

Our study adds to prior research on caregivers’ challenges by examining the relationship between specific challenge-related topics and the ways caregivers seek support on the web. Previous research has primarily focused on either informal caregivers’ sharing of challenges on the web [29-31] or how they seek social support [60] as separate phenomena. In contrast, this study reveals informal caregivers’ support-seeking patterns in relation to various challenges by examining topic co-occurrence. When encountering uncontrollable challenges, such as the care recipients’ behavioral issues and the progression of Alzheimer disease and related dementia, caregivers were more likely to express feelings in their posts. In comparison, when facing practical-oriented topics that require solutions—such as finding appropriate assisted-living facilities and managing financial and insurance issues—caregivers were more likely to ask for advice on subreddits directly. Moreover, when facing complex health and medical issues, caregivers often combined seeking advice and sharing personal feelings. These findings suggest that informal caregivers may adopt different support-seeking strategies depending on the nature of their challenges. However, the relationships between challenge-related topics and support-seeking strategies were either negligible or weak. Future research could further investigate the stress-coping process in caregiving to understand how caregivers cope with their various challenges.

This study also provides evidence for the unique challenges faced by informal caregivers in 2 caregiving contexts. Prior studies have mainly focused on web-based discussions of one caregiver population, either informal caregivers of general older adults [30] or those taking care of people with Alzheimer disease and related dementia [17,28,29,31], without directly comparing the 2 groups. This study compared the topics these 2 caregiver populations discussed, revealing differences in their concerns and support-seeking behaviors. Informal caregivers of general older adults were more likely to discuss practical topics, including care facilities and financial, legal, insurance, and technical issues. In contrast, caregivers of people with Alzheimer disease and related dementia tended to focus on health and medical-focused issues, such as sleep and incontinence problems and Alzheimer disease, and expressed their feelings more frequently in their posts. The findings are consistent with studies emphasizing the complexities faced by caregivers of people with Alzheimer disease and related dementia in health and medication management tasks. For example, a survey by Riffin et al [65] found that caregivers of people with Alzheimer disease and related dementia encountered greater challenges in managing health-related tasks than caregivers of older adults without Alzheimer disease and related dementia. Hence, future research can examine the distinct challenges faced by informal caregivers in different contexts.

Furthermore, the differences in discrete emotions expressed by informal caregivers of general older adults and caregivers of people with Alzheimer disease and related dementia also mirror the premise of the Caregiver Stress Theory [10] that caregiving contexts shape individuals’ experiences. Previous studies have found that caregiving burden, especially emotional burden, can vary across different types of caregivers, especially among dementia and nondementia caregivers [11,13]. Compared with caregivers of older adults, caregivers of people with Alzheimer disease and related dementia face the irreversible progression of functional decline in their loved ones [66] and experience worries and anxiety [13,66]. Our study adds to this understanding by providing evidence that caregivers of people with Alzheimer disease and related dementia tended to disclose higher levels of fear and sadness on Reddit. A similar pattern was also identified through the positive weak correlation between Alzheimer disease–related topics and caregivers’ expressions of fear and sadness. These findings underscore the importance of further investigating the unique emotional experiences of informal caregivers in the context of Alzheimer disease and related dementia care.

The negligible or weak correlations between topics and emotions provide a nuanced understanding of informal caregivers’ web-based social sharing of emotions [36]. Our study found that discussions on the duration of medical care and medical appointments had a complex set of negative emotions, including anger, fear, and sadness. This finding corroborates earlier research showing that dealing with long-term medical care of their loved ones is an emotionally distressing process that often elicits negative emotions [1]. In addition, our study found that informal caregivers tended to express anger when discussing financial issues. While previous studies have mainly focused on the financial burden faced by informal caregivers of older adults, they have often overlooked specific barriers and caregivers’ emotional responses to them [67,68]. Through deep reading, we found that many caregivers felt angry about the barriers they encountered when trying to manage their care recipient’s assets and applying for the power of attorney. This implies caregivers’ dissatisfaction and frustration with financial and legal systems in supporting their caregiving responsibilities.

Notably, this study found a negative weak correlation between the topic of Alzheimer disease and expressions of anger. Previous survey-based research has found that feelings of anger were prevalent in caregivers of people with Alzheimer disease and related dementia [69], and the presence of disruptive behaviors in people with Alzheimer disease and related dementia was positively correlated with their anger expression [35]. Different from existing studies, our findings suggest that when caregivers were discussing Alzheimer disease, they were less likely to express anger or resentment. One possible explanation for this discrepancy is that informal caregivers of patients with Alzheimer disease may develop a heightened sense of empathy and understanding toward the care recipient over time [70]. The increase in empathy may lead to reduced anger emotions and corresponding emotional expression in their online discussions [70]. Another possible explanation is the difference in how emotional expressions were measured in this study compared to prior research. To address these discrepancies, further studies adopting other research methods are warranted to investigate caregivers’ emotional responses and expressions toward Alzheimer disease–related topics.

### Implications

This study contributed in 2 aspects to the literature on the use of OSCs in informal caregiving. First, by illustrating specific challenges and emotional expressions of caregivers of general older adults and caregivers of people with Alzheimer disease and related dementia, this study suggests the importance of examining caregiver stress and coping by considering the role of caregiving contexts, especially the health status of care recipients. Second, this study was the first to empirically examine the correlation between topics and emotional expressions among informal caregivers of older adults. By providing evidence of how informal caregivers express their feelings in response to various caregiving-related challenges, the findings offer a foundation for future research to develop a more nuanced understanding of the patterns of web-based emotional sharing, particularly in relation to the nature of challenges.

Practically, the findings of this study could inform health practitioners and policy makers on how to support the 2 caregiver populations. We found evidence that informal caregivers sought advice and expressed feelings on a variety of caregiving-related matters in OSCs, such as the care recipients’ health and medical issues; dealing with family relationships; and practical topics, such as finance, insurance, and technology. The findings bring to light the needs of caregivers for both informational and emotional support in various aspects, which can inform support interventions and services to meet the needs of the population. For example, addressing financial issues through clear guidance on managing assets and applying for power of attorney could benefit caregivers.

Caregivers of people with Alzheimer disease and related dementia discussed more about the medical and behavioral issues of their care recipients, such as duration of medical care, sleep, and incontinence. This underscores the need for targeted support interventions, such as education and training sessions on Alzheimer disease and related dementia-specific caregiving skills. In addition, the frequent expression of negative emotions, especially among caregivers of people with Alzheimer disease and related dementia, suggests the importance of emotional health interventions. OSCs such as Reddit can serve as a valuable forum for caregivers to share experiences and gain emotional support from their peers. To foster this, OSC moderators should encourage open sharing and facilitate supportive interactions among caregivers.

### Limitations and Future Works

This work has several limitations. First, we focused on the topic distribution and emotional expression across informal caregivers of general older adults and people with Alzheimer disease and related dementia, while not examining other caregiver populations. A potential direction for subsequent studies is to compare the challenges faced by informal caregivers of older adults with different diseases, such as stroke and diabetes, in a more nuanced manner. This can provide stronger evidence for how specific caregiving contexts, such as the health status of the care recipients, shape caregivers’ experiences. Second, this study only investigated the characteristics of informal caregivers’ communication patterns on Reddit, a platform dominated by users from the United States. According to previous studies, informal caregivers of older adults also use other OSC platforms, such as Facebook groups and CaringBridge. Future studies could compare topics and emotions expressed by informal caregivers across platforms to enhance our understanding of the role of different OSCs in supporting informal caregivers.

Third, web-based community users may differ from the broader population of informal caregivers in terms of socioeconomic status and media literacy. Therefore, the findings of this study cannot be generalized to all informal caregivers. This limitation stems from the study’s specific focus on understanding how caregivers use OSCs to share challenges and emotions. Future research could address this by including nonusers of OSCs and comparing their experiences with those of OSC users, providing a more comprehensive understanding of informal caregivers’ experiences across diverse contexts. Fourth, the research methods of this study only allowed us to have a general understanding of informal caregivers’ challenges and feelings. Although we adopted qualitative approaches to analyze themes and meanings of representative posts to complement the results of computer-assisted text analysis, we still cannot infer caregivers’ perceived stress and their actual emotional well-being status. Future research could combine computer-assisted text analysis with other methods, such as interviews or surveys, to have a more comprehensive understanding of caregivers’ experiences and needs.

### Conclusions

In this study, we examined web-based discussions among informal caregivers of general older adults and people with Alzheimer disease and related dementia on Reddit, including their challenges and emotional expressions and their relationships. Informal caregivers discussed a wide range of caregiving-related challenges on Reddit but they varied across caregiving contexts. Caregivers’ web-based messages also indicate a high level of negative emotions, including fear, anger, and sadness. In addition, caregivers expressed distinct emotions in response to various challenges, depending on the nature of these challenges. Taken together, this study elucidated informal caregivers’ actual problems and needs in different caregiving contexts, thereby providing suggestions for health practitioners and policy makers to develop more targeted support interventions.

## Data Availability

The datasets generated and analyzed during this study are available from the corresponding author upon reasonable request.

## Appendix

Multimedia Appendix 1 Representative posts within each topic.

## Abbreviations

API: application programming interface
NMF: nonnegative matrix factorization
OSC: online support community
RQ: research question
