# Supplementary material for: Understanding Challenges and Emotions of Informal Caregivers of General Older Adults and People With Alzheimer Disease and Related Dementia: Comparative Study
Source: J Med Internet Res. 2025 Feb 28;27:e54847. doi: 10.2196/54847 (PMC11909488; doi:10.2196/54847)
Supplement: Multimedia Appendix 1 [file jmir_v27i1e54847_app1.docx]

**Appendix 1. Representative posts within each topic**

**Topic 1: Express feelings**

**#1 Title: So frustrated and sad**

“Hi all. I just need to talk about this in a place where people will understand.

I take care of my auntie during the daytime every day. There's a nurse who comes at night. I love my auntie and we have always gotten along well. However this week I feel like I couldn't recognize the aunt I once had such deep and important conversations with. The disease has made her more and more childlike and this week was the worst yet, I feel so hopeless knowing with 100% certainty that it will only continue to get worse.

She has developed echolalia, since she cannot remember a lot of words she will just repeat what I've said, and she has a few phrases and noises that she repeats like a broken record throughout the day.

Today she said the same phrase literally over 200 times, it was like she got stuck. In between those 200 times she was repeating other noises and phrases. We did not have a single conversation all day. Usually I could get her to say if she's hungry or wants to watch TV, basic things but still small conversations where we understand what each other means.

Obviously the repetition is annoying but I can handle annoyance. It goes beyond that, it is haunting to see a person you love disappearing right before your eyes, when she kept saying that phrase it was like her spirit wasn't even there anymore, she was just making noise because she couldn't figure out how to stop.

The part that is making me so upset, is that I will forever hear her voice in my head saying these things, instead of hearing her say the kind, profound, and interesting things we used to talk about. It feels like my memories of her at her prime are being replaced by this version of her. And eventually this version of her will get replaced by a worse version of her. Even as I'm typing this I can hear her saying it over and over again and it makes me feel sick, I can't describe it in words but I hope you know what I mean. That wasn't her yet it WAS her and the dissonance between the two versions is what's turning my stomach. I did not let it show to her but when I got in my car to leave I couldn't hold back crying. I always knew the disease would bring her to a point like this but there is nothing that could have prepared me for how it would happen or how I would feel. I called my cousin/her son who lives far away and he wants to ask about med changes at the next doctor's appointment. As if it's that simple. I'm not opposed to it but the way he said it, made it clear that he didn't really understand. And I felt very alone after that call.

I do not resent taking care of her or intend to stop but I admit I feel very jealous of the family members who live far away and are able to keep the good version of her in their minds forever. Even a short visit here and there doesn't erase the memories of the real her, I feel like it's only me who sees her every day who is going through this feeling of losing her in real time and already feeling haunted.

Anyway thank you if you read this far, this disease is so cruel. I know we will probably have some more good days here and there and will try to look forward to those but this is definitely a turning point for me. The mourning process truly began today.”

**#2 Title: My grandmother refuses help from anyone but her son, but her son has cancer**

“Does anyone have a parent or other older loved one with bad health issues who is always feeling down about it? Mine has been dealing with some serious health problems and an endless string of doctor visits and medications that haven’t been of much help. Understandably, she is very upset about the situation. She often says things like she is so tired of feeling this way, she’s a shell of who she used to be, she doesn’t want to go on like this anymore, she doesn’t know how much time she has left, and so on. Makes me so sad because 1. I love her and the thought of losing her terrifies me. 2. I feel like there’s nothing I can say or do to make it better. Usually I just say I’m sorry things have been so tough, I am always here to help however I can, and hope things get better soon. But it just seems so hollow and unhelpful and I feel like a broken record. She also has a big phobia of dying and I don’t know how to be of any comfort there either, since I feel the same way (even though I’m in my 30s). Has anyone else dealt with something similar? Is there anything I can do or say in this situation? She’s been there for me, and now I wish I could do the same.”

**Topic 2: Duration of Medical Care**

**#1 Title: Last weekend my dad was fine**

“100% independent. All I did was come mop the floors for him. He has a bad back,so very slow. I would go for weeks without calling (I live 2 hrs away but go down every week to fortnight to see him)This Saturday I did a wellness check because he wasn't answering his phone. Apparently he was hot, so he decided to sleep in the bathtub (delirium) and couldn't get up. His phone was accross the house absolutely no where near him. His elbows are all bloody from where he has tried to get up. The bath faceut is bent. But even then, when I got there he said he was fine and I didn't need to come in. I had to break in and climb through the bathroom window. Literally so much faecal matter, must of been there for 12-24hours. didn't want me to call an ambulance. didn't want to go to hospital (once the paramedics were on site). So stubborn.Maybe the delirium is to protect him from knowing too much too. Since he's been in hospital he's just been in an adult diaper and literally continuously laying in his own poop. I didn't realise things went downhill so quickly with the elderly. I know it's an infection causing the issues but it's just so confronting.The doctor says I'm preoccupied with their reputation for kicking patients out and says the goal is to get him back to independent, but boy. That feels so far away right now, when I can barely enter his room from the stench.Edit: He is home! He had an unknown source of infection which caused delrium. He spent 5 days in hospital and walked himself out! he is very very low on energy and not 100% so we are monitoring super closely and I am visiting everyday but hopefully after a few weeks at home he starts to feel himself again. Very very lucky and counting all our lucky stars. Plus a big wake up call that we have to do things a bit differently from now on.”

**# 2 Title: Near the End**

My mom is almost gone. She had a stroke type thing happen on November 1st. They wouldn’t say it was an actual stroke, because the CTScan didn’t pick it up. They said they got some enzymes of telling there was heart strain also. They won’t call it a heart attack. I think it was actually somewhere there is external bleeding and that made the heart not pump and did some damage to the brain. She hardly could talk and I did put her on hospice just to let her go without having her go through figuring out where the bleeding is (probably cancer) and then have her more disabled as before.

Unfortunately right after we got her on hospice she started to go down hill fast. Monday they only gave her a week or two, today they only give her a day or two. Turns out she has Covid, we have Covid too, I think we got it from her, which she probably got from the spread in the facility.

I don’t know how to feel really. I feel I let her down. I keep flashing back on getting her to go to assisted living last year. She said “come on just take me home”. I wished I did. Maybe, just maybe she wouldn’t of progressed so fast. She was speaking in full sentences at the time and before the stroke, she was just using a few mumbled words.

Maybe I could be still spending time with her at her house doing puzzles and fixing her meals. Maybe her dogs would still be alive. They past this year, one had kidney failure, one had a tumor that enveloped his whole abdomen. I dunno. I know this is all called ruminating, and it’s bad, but it’s just where my mind is right now.

She was just getting a pump for her morphine when I last saw her, I held her hand I told her it will be alright and I love her. She didn’t respond. I did see a tear kind of form in the corner of her eye.

I don’t know what I’m going to do without her. All my life I tried to just keep her safe, from my alcoholic dad, my brother and sister draining her bank accounts, her losing her mind slowly. I got some of her dreams fulfilled, like seeing Grand Canton, Banff, Alaska. Taking her back home to see her old house and farm.

I just don’t want it to end, I wish I could just whisk it all away somehow and make her better again. Maybe even a few minutes so she could have just one more cigarette and tell me a story of how I did something that I actually didn’t do, but don’t fight it and just let it be, let her think it’s what happened and she laughs. I laugh. Then she’ll tell it again in a mother couple of minutes while lighting up another smoke.

**Topic 3: Aging and Declining Health of Parents**

**#1 Title: Two Generations of Aging Parents**

“Do any of you have parents aging at the same time as your grandparents? Due to our family’s longevity I (40F) have parents and grandparents all aging. We’ve noticed some very mild cognitive issues with one of my parents over the last few years which could be related to other health issues, but recently their parent took a turn and began to more rapidly decline. Ever since that time our parent seems to have more significant signs of cognitive decline themselves.Note: None of us live in the same state, so while I talk and message everyone regularly, I don’t often see people in person. Our parent was not particularly close with their parents (my grandparents) and seems okay when we talk about my aging grandparents, but I can’t help but wonder if there is some added stress or depression occurring. My parents are not acting as caregivers and are not local to my grandparents.I would like to hear your stories. Have you experienced aging grandparents as a stressor so concerning that your parents began to present with/accelerate issues too? Tell me about your experience.”

**#2 Title: How to cope with depression over aging parents?**

“My parents are still living independently, but after my dad's (minor) stroke this year, I've been emotionally supporting my mom a lot, since we've always been very close. I've been depressed over the last month, and I've realized that it's over knowing that my parents' health is going to continue to decline and their personalities will change and eventually I won't have them anymore, at least not as I know them now. I'm new to this kind of grief, and I don't know how to cope with it. I know it isn't sustainable to feel this way every day, but what can I do? How do you find comfort?”

**Topic 4: Seeking advice**

**#1 Title: Help please**

“So, my father has no health insurance and has been denied state insurance because he isn't "disabled" well he can't get him deemed disabled until the state sends him to one of their doctors to be seen, which is taking forever. He is in very rough shape. Confused all the time, forgets to eat, forgets to bath, is starting to ask really strange questions. Me and my fiance both work full time. We're starting to worry he needs more help that we can offer. I've called a few places and pretty much received no help. Is there any program that will help? He's getting worse every few weeks. I worry that I'm going to have to take him to the ER just to get help but I know they really can't do anything for him either.”

**#2 Title: My grandmother refuses help from anyone but her son, but her son has cancer**

“**I need advice.**

My grandmother is 91 years old and cannot function on her own. I moved down about 2 months ago to help after a fall, but did not know her prior to that.

She is peeing on herself consistently, refusing to wear depends, and threatens violence when she doesn't get her way. The only person she will listen to is her son.

I told my grandmother that a nurse will be over to help bathe her tomorrow. After being screamed at, I told her that my uncle cannot bathe her after every accident, and i cant stand the smell of her urine.

I don't know what to do. I was so desperate. I thought maybe if I made it about me, she might want to want to be wiped down properly, but it just made things worse. I don't know what I'm doing and I can't stand it here. My uncle has stage 4 pancreatic cancer and is barely functional himself. He has two young children and a job on top of that. He cannot help her in the ways she wants.

I am desperate. What do I do?”

**Topic 5: Finances**

**#1 Title: Selling my father's house and car when bank refuses POA (Canada)**

“Like the title says, I need to sell my father's house and car as he is now in a nursing home and those bills are draining his account but the bank he uses refuses to accept his POA. I was able to set up online banking to take care of his bills but not sure how to proceed from there. I never owned a home so don't know much about the paperwork/contract involved. So here are some questions:

-Do I actually need to have a POA setup at his bank to sell his home or can I sell it then just deposit the money in his account and pay off the debt?

-What else can I do to get POA over his account? I think I saw someone mention that you can try another branch altough I doubt it would change the outcome.

Any help on this is appreciated.”

**#2 Title: Help!**

“This is going to be long, so be patient with me! So we were able to get POA, thank the lord! But I feel like the attorney swindled us into getting other paperwork we didn’t need… my MIL is pretty well off, has 370K in savings. A paid off house that is just sitting (since she’s lived with us since 11/21) and the lawyer told us to do an Irrevocable specials needs trusts, in addition to the living trust. Which we would need to pull her money out 10K at a time via cashiers check, husband would create a new acct to deposit and then transfer to a trust set up for her? To make it look like she’s broke so Medicaid will pay for her facility when the time comes. I had questioned it but TBH my husband and I were a little confused. This paperwork cost an extra 4K. (Our total being $9220, yes it’s a lot.) she has a net income of 7200 and when we were browsing a memory care facility will cost 5-6500K. Which is fine, she can afford it.(she has no other bills) if we did his route Medicaid would take her full $7200 income anyways?! So I’m thinking, why would we go through all this trouble to move her money if they’re going to take her monthly income anyways?! Idk hopefully someone can help me understand… or maybe he was good at up selling us”

**Topic 6: Memory Care Facility**

**#1 Title: Memory care facility**

“Has anyone had the experience with a memory care facility starting to feel like a cash grab? My mother has been in a AL/MC hybrid floor maybe six months now. Maybe a little more. When she moved in she was at the lowest level of care on that floor. Now she's at the highest level and increased the monthly cost. Just this week they said she's been "evaluated" and is in need of going to the full on memory care unit which costs more. I'm not there very often but I'm just not buying it. I don't see the big change that would trigger this.”

**#2 Title: Memory Care Facilities, Northeastern US**

**“I’m desperate to find a memory care facility for my parents but it‘’s so overwhelming.**

For some background, my father has Alzheimer’s (although I don’t think it‘’s an official diagnosis) and my mother has early dementia. She tries her best to take care of him but she needs help. We have caregivers coming by 5 days a week (for a couple hours each day) and my brother, who is local, checks in on the weekends. This environment is not sustainable so we’re trying to find a memory care facility in Vermont (where they currently reside), Massachusetts or Rhode Island (nearby family could visit). We do not want to separate them. We keep coming across waitlists. Is there truly nothing available anywhere? This is such a hard process to navigate. I’d appreciate any tips. Thank you!”

**Topic 7: Alzheimer’s**

**#1 Title: How hereditable is early onset Alzheimer’s?**

“I’m not sure if anyone here knows, but IÃ¢Â€Â™m not sure where to ask.

My grandma has early onset AlzheimerÃ¢Â€Â™s. I would like to know how likely I am to develop it”

**#2 Title: What is Alzheimer's like for the person who has it? Do they realize how serious it is?**

“Alzheimer's disease affects individuals differently, and the experience can vary depending on the stage and progression of the disease. In the early stages, individuals with Alzheimer's may have awareness of their cognitive decline and memory loss. They may recognize their difficulties in remembering names, and events, or finding the right words during conversations.

However, as the disease progresses, individuals with Alzheimer's may have increasing difficulty understanding or accepting the seriousness of their condition. They may experience challenges in recognizing their own cognitive decline, and their ability to fully grasp the implications of the disease may diminish.”

**Topic 8: Sleep and Incontinence**

**#1 Title: Overnight Incontinence solved with adjustable bed?**

“We used to wake up every two hours to go to the bathroom like clockwork. Eventually we would be so tired of not getting enough sleep that we would sleep through a pee and wake up 4 hours later.

Recently we got an adjustable bed to help avoid bedsores and we have been getting MUCH better sleep. In fact we don’t wake up at all to use the bathroom. We still use the overnight pad to absorb urine but we can still stay in bed (COMFORTABLE) for a full nights rest. Seemingly only peeing once.

I have a feeling that we are able to do this because we sleep with our feet and head raised up. Maybe the new positioning helps us not feel the urge to go. Almost like kinking a hose(?). We don’t sleep at an extreme angle, we are basically flat, but the slight change in position seems to be working for us.

Has anyone else had similar success?

* as a side note, our bed has become way more comfortable.”

**#2 Title: Sleep**

“My grandma has advanced dementia and she is having trouble sleeping. It's like she's fighting it. She will tell you how tired she is all day then refuse to sleep at night.she sleeps at most 4 hours a night. Even if we keep her up all day she still won't sleep. Help please”

**Topic 9: Dementia**

**#1 Title: Progression of vascular dementia**

“Question for those who have a LO diagnosed with vascular dementia:

How long ago were they diagnosed?

How has the illness evolved since then?”

**#2 Title: Dementia “not feeling well” all the time**

“My dad with dementia (75) tells us, for years now, all day long that his ears are ringing, everything hurts and he doesn’t feel well. I’ve had him in the emergency room multiple times and outside of his blood sugars being too high (he’s diabetic and doesn’t eat properly due to his dementia,) they say he’s fine. Of course, he won’t submit to other testing, so there could be a cancer lurking or something we’re not aware of, but the doctors are telling me it’s just dementia.

Anyone else deal with this..? I am patient with my dad 98% of the time, thanks to having help from 2 wonderful aides, but it does get frustrating listening to the constant complaints. Plus, I worry that there is something I’m missing in terms of his health, even though I know his dementia means he won’t understand/agree to treatment for other issues anyway.

The constant negativity wears on me. He does take several meds for mood/depression by the way.”

**Topic 10: Assisted Living Facility**

**#1 Title: Average time is assisted living**

“I read a statistic that said the average stay in assisted living is 22 months. This seems low to me. I’m curious to know how long some of the aging parents referred to on this page have spent in assisted living. Anyone willing to share? Thanks”

**#2 Title: Assisted Living Questions**

“I just came from a doctor's visit with my mom in which the doctor stated that neither of my parents can live independently on their own. He said that we had three options: a family member cares for them, a caregiver moves in with them, or they move into assisted living. Neither myself of my brother feels like we can provide 24 hour care for them as we both have jobs in much larger cities therefore we were trying to decide between assisted living and having a care giver in their home with them. My parents are concerned however that someone will take the house if they go with the assisted living options. What I am wondering is, is this true?”

**Topic 11: Nursing Home**

**#1 Title: mother scared of being put in nursing home**

“Anyone have a parent that is scared of being put in nursing home?

We are planning to sell our house and move into a condo or smaller house. But she gets so nervous and scared that I am putting her in a nursing home.

Any tips ?”

**#2 Title: Right of rescission for nursing home decision?**

“A relative recently suffered a stroke. He had been hospitalized and case workers (hospital and nursing home) arranged for short term 100 day in-patient rehab at a local nursing home. His wife now desires to caregive for him at home and wishes to undo the agreement she made last week. Is there a a time within which she is able to do this? We have been told 30 days, three days, etc. Please help.”

**Topic 12: Medical Appointments**

**#1 Title: First Doctor Appointment on Tuesday**

“So my LO walked out of his last Neuro Appointment so we set another one as a Primary Care Physician on to not alarm him so much. What if he decides he is not going to go? Our plans are pretty freaking Draconian to try to get him to the doctor if he refuses. He walked out of the Neurologist appointment we set with a great one and is delusional about it. He has not even been to the doctor about any of this.

So we got him a Primary Care appointment to get a checkup and physical, bloodwork the most common stuff but now he is resisting. These appointments take a long time to make. He walked out of the last one and he might walk on this one. I hope not and I plan on being calm as shit but then what after? We need to get him diagnosed right now he is in like stage 4 and never been to the doctor and going loose. FML!”

**#2 Title: I think my mom has earpyonset something...**

“Her memory has gotten really bad lately like the last few years. I just yelled at her to make a doctor's appointment because I realize she needs to start meds for this. Well she refuses to go to the docter, I said make an appointment by Friday or I am doing it for you! Can I even do that? This is fucking me up bad and I'm not sure what to do. I would greatly appreciate advice.”

**Topic 13: Cell Phone & Devices**

**#1 Title: Google smart devices for elderly with memory challenges?**

“Hi, I generally despise "always listening" smart devices because I value my family's privacy. However, I'm looking for a simple way for my mom to find her phone when she loses it, and it seems that something like a Google Nest may be the thing. (I tried a Tile that we would hang on the wall so she could double-click it to find her phone, but that requires the Tile app to be running, the phone not to be in Do Not Disturb and especially the first is hard to ensure.)

So, if Mom could just say "Hey Google, find my phone" and it would ring her phone, that would be a win.

So then I wonder, what else could it do for her? Could it connect to a music subscription service and play music for her? (Hey Google, play some show tunes?) If you have elders with Google Nest/Home/similar devices, what do they use them for. Mom has Mild Cognitive Impairment (verging toward not so mild, I suspect) and is bad with technology, which is why she can't just open her laptop and go to the google find my phone page despite me making a shortcut for her.

I'm leaning google rather than amazon because it's an Android phone, but open to other options.”

**#2 Title: Looking for a phone that might not exist - any advice?**

“My mom, even when she puts her hearing aids in, can barely hear her phone. She uses it on speaker all the time because she believes that helps.She really likes having a landline with a wireless handset, but she's now reached a point where she needs something simple, with BIG buttons, that has amplified sound. (she accidentally blocked me the other day...yes, it really was an accident. She was sure someone came in and sabotaged her phone). She can't do a cell phone.So, does anyone know of a landline make/model that is like this but the handset is wireless??Thanks in advance!!(added some clarity)”

**Topic 14: Parent (mom)**

**#1 Title: Mom is so mean**

“Hi everyone! My mom was diagnosed almost 2 years ago and getting worse so quick.

We finally found an amazing caregiver to be with mom. Our caregiver is kind, actually cares about my mom and is trying so hard to make mom happy.

The problem is my mom is so mean to her. She ignores our caregiver, screams at her, accuses her stealing her things. When I hear all this screaming I of course go and see what happens and as soon as mom sees me she smiles and smiles at our caregiver and treats her kindly and jokes around with her. And as soon I leave, all the screaming starts again.

Does mom know what she is doing? It seems like a little child who is being mean to someone but as soon as their parents walk in they change their attitude.

We have tried to tell mom to be nice and don’t say hurtful things but obviously she does not retain it long enough.

Anything we can do?! I really hope this caretaker does not leave :(”

**#2 Title: How would you choose where to live? We’ve got two aging mothers in two different parts of the country.**

“Don’t know if anyone can relate: Husband (M50) has aging, disabled Mom in MidWest. My (F50), mom lives in California (where we live now) - she’s not disabled but definitely into guilt tripping me about staying here, close to her. She’s done this as long as I can remember and it’s quite frustrating. She’s divorced for 25+ years. Her son, my brother, lives 1 hr away from her.Both Moms are about the same age (early 70’s). Husband’s father died last summer, leaving MidWest mom a widow.MidWest mom is willing to go in on a home with us - she would live in an ADU behind the main home where we would live.California mom (mine), wants us to stay, but our home options, strictly due to cost, are 2-4 hours north/away from her and it doesn’t solve our dilemma with husband’s MidWest mom. CA mom is unable to go in on a home with us (we’re not expecting her to, just stating the facts). We’re stuck not wanting to rent here forever, want to buy a home, take care of husbands mom but California mom will freak out and readily inform me what a mistake that is and that she will Â¡Â°have no one (the trust level between her and my brother is on the weaker, wonkier side. I’m the elder daughter, so she’s always trusted me way more and leaned on me for pretty much everything).I know this is a lot. It’s just all so depressing and overwhelming to figure out. I was also tempted to offer to take CA Mom to the MidWest, but then that leaves my brother with no one except his two young daughters.Thank you for reading and/or advice.”

**Topic 15: Parent (dad)**

**#1 Title: dad's the one with Alzheimer's but I miss my mum**

“My dad has Alzheimer's. He lives at home with my mum who is his primary carer. I am normally good at finding the positives but I'm truly struggling lately. It's so hard to remain patient and positive for a man that was once my father but now bears very little resemblance to him.

I have a baby, mum looks after her one day a week. Last night they came to babysit but dad, obviously confused about being anywhere other than home, repeatedly woke her and we ended up cancelling our evening out so mum could take dad home.

I want to go out with my husband. I want my mum to be able to babysit which she loves doing. I want to spend an evening with mum. I want to have dinner with her and watch a film. But we can't because dad needs to be at home and needs so much of our focus and attention there is little left for anyone else.

I miss my mum.”

**#2 Title: My dad is old and depressed**

“I need help me (41f) with my dad (75m), I have been working on my depression for about 15years. It's taken a long time and a lot of help. It's obvious that my dad has depression, but of an era were it doesn't count, even though he has supported me at every step. He is so miserable all the time, my mum worries every time he leaves, he will "fall" under a bus. How can I convince him to seek help?”

**Topic 16: Parent (mother)**

**#1 Title: My sister won't take care of my mom!**

“Hello everyone: My mother is in her 80's and fairly functional with activities of daily living. She did have an operation back in October, but it has not affected her ability to eat, speak, walk, or use the restroom. The operation was a shock and happened about two weeks after she moved in with my younger sister's family. It was scary for all of us, but I think it was too much for my younger sister to handle. I offered to have my mother at my house Tues through Thurs each week to take some of the stress off my sister, but now I feel I am being taken advantage of. I work a school year schedule, so now my sister thinks I should have my mother all the time because I'm not working. My sister worked 3 days a week the entire year, and I honestly made the plan to help all of us, not just her. It is very hurtful to me that she will do anything to get rid of our mother for a few days so that she and her family can have the run of the whole house. She keeps offering up my services because I am off work for the summer. I am having both bathrooms remodeled later this month because the shower that was built for my mother at my sister's house is a death trap, and she refuses to use it. So, she has a very strong odor when she comes here, which creates a ton of extra work for me because I have to wash everything every week. I also might have jury duty starting next week, and I will not be here if I am chosen. I do not want my mother to know or hear that her child does not want to care for her, and my other sister and I have told her three different times that she can stay here if she changes her mind. I feel that I am being used by my sister to get out of doing what she said she was going to do, which was care for our mother in her home. I have honestly thought of saying I had COVID so I could have a break too! I think my mother just refuses to admit that she made a mistake when she moved in with sister's family. I do not pressure her to move again, never participate in bashing my sister's lack of cleanliness, or complain about the lack of communication from my sister about the schedule. I am trying to be the bigger person here, but I'm just tired. Any help or advice would be greatly appreciated. Thank you!”

**#2 Title: Mother showing manipulative behavior**

“Any one dealing with this? My mother was diagnosed wit AZL after a long time with dementia. There is meltdown with the relationship between me and my sibling that goes way back, and partially due to the lack of care she provided during times of my parents illness (my father also has dementia). I recently found out an in-law was granted POA over my mother years ago, but I was never informed. My mother during this time has reached out to me so many times I cannot count, wanting to go into assisted living, claiming my sister will not help. The last time I was so frustrated b/c my mother cornered me about it when I was there to take her to lunch, and rather than go to lunch I got bombarded with how noone will help her. I emailed my sister about it, and needless to say it did not go well, and I was informed her husband has POA. So my mom recently called me b/c I have to remove my self from this....it is toxic for me, and she had not heard me. I politely explained that since she gave POA to my brother-in-law there is nothing I can do and that I hired an attorney b/c I do not know what else to do. If my mom granted it during dementia it is questionable, and since the communication is broken between my sibling's family....I have not idea what is going on. The response was not good, she hung up on me (the call lasted less than 5 minutes) then called my sister to say I verbally abused her on the phone, had to take a valium (she has been addicted to this type medication for years) and to ask that I not call her again. This is not surprising as mom mother's behavior has always been somewhat manipulative with a need for attention. It is a nightmare, and needless to say I am ready to leave the situation 100% for my own peace of mind. Thoughts?”

**Topic 18: Grandparents**

**#1 Title: Grandparents-what to do when the one in charge of medical care is unable to care**

“My grandma and grandpa live together with a nurse that comes by once a week. my grandpa has advanced Alzheimer's and cannot walk by himself and cannot feed himself very well. My grandma is also struggling to walk by herself and I am not sure how they are currently able to live without an in-house care giver.

What options exist to take care of my grandpa if my grandma and becomes unable to? Family caring is not an option as the house I live in has too many steps for him to get into and our house cannot accommodate a wheelchair. What could I do for emergent care for him if my grandma was admitted to the ER? I am also not sure what I need to do to take care if my grandpa daily. I am a new mom, but apparently my mom and grandma have decided that it would be my problem to solve if the time came.

My grandma does not want to discuss this issue further because it upsets her and my mom ( their daughter) is the same way...”

**#2 Title: Grandma is incapable of caring for herself but refuses to hire any help or move**

“My grandma (79) has had a ton of health problems for decades. She relies on my grandpa (80) to do everything for her (feed her, push her wheelchair, etc). She spends all her time yelling at him and I can see it is taking a toll on him. She will scream at him for hours and he is struggling to take care of himself (recently had surgery for cancer) and her at the same time. Their children agree with me that we need to hire someone to care for grandma but she refuses. My grandparents have the money for it but are extreme cheapskates so grandma will not allow it and is refusing to acknowledge that grandpa is not at his best. Their children cannot afford the level of care she needs. We have also tried to talk her into moving into a care facility but she refuses to leave her house. They live in the hills in a fairly remote area. Additionally, both of them are horrible drivers and should not be driving let alone driving down the hills to the city below. The majority of my family cannot stand to be around grandma but she’s stubborn and refuses to accept care from anyone but grandpa. Any advice on how to handle this? We would hate to go behind her back and force her into a care situation but we don’t know what else to do.

Edit- I am also curious as to whether her anger outbursts may be a sign of a bigger issue. She’s always been an angry person but she’s been more aggressive as she ages.”

**Topic 19: Long Term Care Insurance**

**#1 Title: I need help finding long term care insurance**

“My husband has just been diagnosed with Alzheimer's, so I'm beginning a search for long term care insurance. We live in Texas. Does anyone have any resources? I searched online, but it seems the results I'm getting are for health insurance, or I find that I can't get it with the diagnosis. Thanks!”

**#2 Title: Long term care insurance**

“I'm a 31 y/o female currently caring for my 82 y/o grandma.Long story short- her kids (my mom and uncles) are either incapable of caring for her or unwilling. Cousins - same. So it's just me.After seeing how failing to plane properly for your last years is a burden to those who are even remotely able to help - I'd like some advice.Is it too early to look into long term care insurance?My husband and I are both early 30s and expecting our first baby.We only plan on having one and I sure as hell refuse to put this burden on them when I get older. I'd rather my last years on earth be those where they can enjoy me rather than worry about my care as much as possible.”
